# Exploring the Impact of Pre-course High-Fidelity Simulation on Professional Socialization of Medical Students in Emergency Medicine Internship Rotation—A Qualitative Approach

**DOI:** 10.3389/fmed.2022.933212

**Published:** 2022-06-30

**Authors:** Yu-Che Chang, Nothando Sithulile Nkambule, Shou-Yen Chen, Ming-Ju Hsieh, Chung-Hsien Chaou

**Affiliations:** ^1^Chang Gung Medical Education Research Centre, Taoyuan, Taiwan; ^2^Department of Emergency Medicine, Chang Gung Memorial Hospital, Taoyuan, Taiwan; ^3^College of Medicine, Chang Gung University, Taoyuan, Taiwan; ^4^International Graduate Program of Education and Human Development, National Sun Yat-sen University, Kaohsiung, Taiwan; ^5^Department of Thoracic Surgery, Chang Gung Memorial Hospital, Taoyuan, Taiwan

**Keywords:** high-fidelity simulation, professional socialization, emergency medicine, authentic learning, community of practice, zone of proximal development, internship

## Abstract

**Background:**

Medical students in block clerkships constantly adapt to new environments and learn to interact with new people as they rotate between specialties. This frequent change potentially limited interns' opportunities for participation in real clinical practice. The aims of this study were to explore interns' conceptualization of their learning opportunities and experiences in the workplace during an emergency medicine (EM) block internship. In addition, the study also explored how participating in the pre-rotation high-fidelity simulation (HFS) orientation influenced interns' perception of their transition, participation and learning experiences in the real EM setting.

**Methods:**

We implemented a newly developed pre-EM rotation orientation curriculum for interns. This orientation took place on the first day of the 2-week EM internship rotation. Two focus group discussions were held after each simulation training, one immediately after simulation to understand the students' perception and the educational impact of this activity, the other at the end of EM rotation to explore and compare their roles and perception in both simulation activity and the real clinical practice. A total of 151 seventh-year medical students enrolled in the pre-course HFS and *post-hoc* focus group discussions between 2017 and 2019. We applied thematic analysis to systemically identify, examine, and construct themes.

**Results:**

Four major themes were constructed from the data; 1. Challenges in finding authentic learning experiences within the context of emergency medicine; 2. Effectiveness of the pre-course HFS 3. Limitations of EM internship rotation curriculum and pre-course simulation. 4. Suggestions for EM block-internship curriculum reforms. Our study's key findings indicate that pre-rotation orientation HFS activity, which offered a psychologically safe space for students to explore facets of EM and gain a contextualized understanding of the emergency work culture and environment, was essential for enhancing students' ability to identify and maximize practice affordances in real workplace.

**Conclusion:**

Simulation, facilitates interns' negotiation of legitimate peripheral participation opportunities as they transition into the EM community of practice during their block internship rotation; putting students at the center of the learning process.

## Introduction

Medical Internships constitutes of all the learning activities provided to medical students in their final years as they transition from medical school into the workplace (1–3). While internship rotation curriculum remains a timely and cost-effective way to present a general overview of each specialty and introduce students to the different medical specialties (4), its effectiveness in providing interns with adequate meaningful learning opportunities to meet their learning requirements has been subjected to scrutiny (2, 5). The major concern for scholars has been how to enhance students' learning experiences during block clinical rotation (2, 5–9). A growing body of research indicates that poor understanding of the work environment, culture and practices of each specialty within the short-period of block internship rotation limits students' opportunities for meaningful interaction with members of the community of practice (6, 10, 11). Students' participation and involvement in each clinical environment depends on these knowledgeable members (10, 12–15). Without these interactions, the likelihood of students to exhibit high levels of anxiety, feelings of marginalization, perceived stress and overall lack of readiness for practice increases (1, 6, 16–19). Due to this, it is necessary that we provide students with support during this period as they move through multiple specialties during their rotations (13, 19, 20). In light of this necessity, we developed a novel pre-Emergency Medicine (EM) rotation orientation curriculum underpinned by simulation-based education to enhance interns' transition process into the EM.

Accumulating evidence indicates that simulation-based education integrated into health professions' students' clinical rotation can be used to augment block rotation (21–23). Simulation-based medical education (SBME) has emerged as a way to help students' maximize their block clinical learning and development experiences (8, 24). A literature review of the role of SBME indicates that it enhances medical students' skills acquisition and scaffolds medical student's professional development, through affording students with a variety of practice opportunities (21, 25). SBME can also prompt medical student's clinical reasoning and enhances their clinical judgment skills through reflective thinking (23). Previous studies indicate that students who participated in pre-clerkship and pre-internship simulation-based education improved their knowledge, technical skills, confidence, were more satisfied with their learning experiences and likely to be trusted by their supervisors and patients (26–28). Based on this, scholars propose that simulation increases trainees' opportunities to participate in the clinical setting (8), thus could potentially help with the process of transitioning into workplace (27) and transitioning into internship, especially under constrained resources (25, 28).

Nonetheless, such research mainly focuses on how simulation provides additional opportunities for students' hands-on practice (29, 30) rather than the role of simulation in orienting medical students to understand the various clinical environments that they rotate into as they move across specialties. It is forward thinking to explore how interns conceptualize the role of high-fidelity simulation exercise conducted before block-internship rotation in enhancing or marring their experiences as they transition into the workplace learning. In this study, taking the EM rotation and learning context as an example context, we illustrate how simulation can be used as a pedagogical tool to orient students before rotating into a new specialty. The EM workplace is a unique clinical setting that combines undifferentiated patient care with varying levels of acuity thus provides medical students with countless opportunities for learning and applying multidisciplinary skills and knowledge to manage cases in an efficient manner (31). However, learning in such a busy environment with high uncertainty has its challenges (32). Literature indicates that medical students do not have adequate patient encounters in their EM rotation or interactions with supervisors (9, 33, 34). This indicates other forms of non-clinical teaching is needed to supplement EM internship rotation (2, 33), especially simulation related learning (4). Our basic assumption was that in addition to affording students with opportunities for hands on-practice, simulation would provide an interactive educational activity for students to familiarize themselves with the clinical environment, model of care and also the team dynamics found in the EM community of practice. By transferring some of the interactive experiences gained during simulation activity, medical students will have imperative experiences transitioning into the EM workplace during their internship rotation. This could be considered as the hallmark of the professional socialization process, an essential part of adjusting into a new work culture and environment (12, 13, 27).

Despite the wide acknowledgment of the importance of simulation in supporting medical students' learning process, there is paucity of literature on the effectiveness of simulation in shaping the transition and socialization process of medical students in short-term rotation (8, 25). Additionally, the current research on students' learning experiences during their EM internship rotation is also limited (4). The role of simulation in enhancing interns' transition into the EM internship remains to be explored (25). Based on this, it is imperative that we conduct a study to explore the nuances of interns' workplace learning experiences in the EM setting. Given that SBME can be tailored to the context and specific needs of individual students (35, 36), we propose that it can be used to introduce students to the model of care and practice for different specialties. Hence, our second aim was to investigate the potential influence of high-fidelity simulation exercise conducted before rotation in shaping interns' perception of their transition into the EM workplace, meaningful learning opportunities and experiences. Results on the role played by simulation in enhancing or marring those experiences contribute to creating an understanding of how we can use simulation as an orientation tool to prepare students to maximize their learning experiences even in complex, multidisciplinary specialties that require broad range of skills like the EM. Such specialties are widely known for being difficult for medical students to transition into during internship rotation (9, 33). The study addressed the following research questions:

1) How do interns conceptualize their learning opportunities and experiences in the workplace during EM block clinical rotation?
2) How does the experience of attending a pre-rotation high-fidelity simulation influence interns' perception of their transition, participation and learning experiences in the real EM internship rotation?

## Materials and Methods

### Theoretical Background

**Figure 1:**
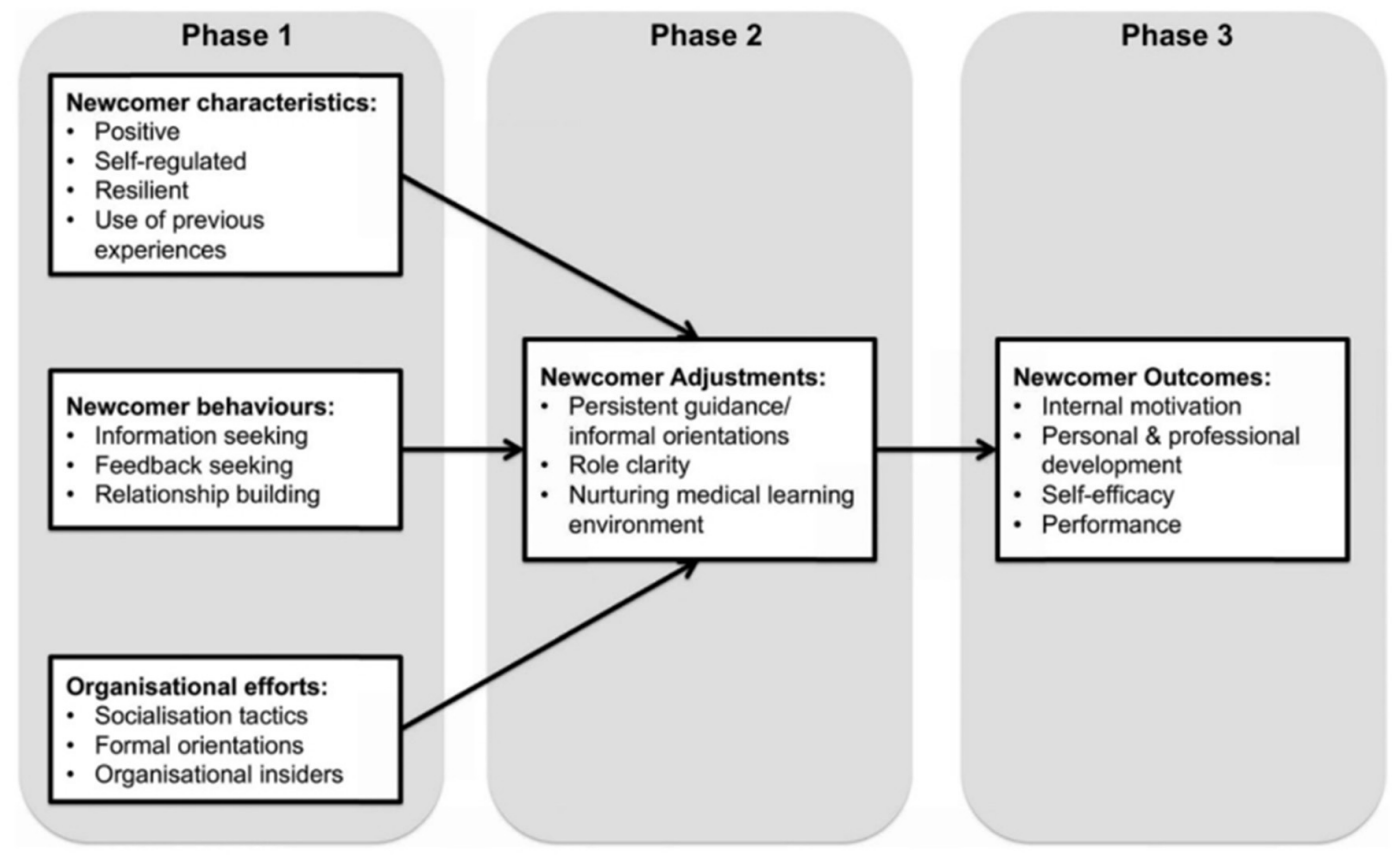
The organizational socialization theory by Bauer and Erdogan (12) modified by Atherley et al. (13). (Used by permission of the authors).

Professional socialization is a “continuous, interactive and transformative” process that takes place when an individual (i.e., novice or newcomer) adopts the culture of their respective profession (27). The organizational socialization theory, conceptualized and modified by Bauer and Erdogan (12), captures the essence of this definition. The theory highlights three factors that are essential to newcomers' socialization process; new employee's characteristics, new employee's behavior and organizational efforts (12). These can be divided into three phases shown on Figure 1 below.

The premises of this theory are supported by literature indicating that interaction and relationships between newcomers and organizational insiders' in the workplace; a product of newcomer characteristics, behavior and organizational insiders' acceptance are key to newcomers' adjustment (37–40). From the medical professional socialization perspective, interactions between newcomers and other members of the community of practice facilitates proper supervision and scaffolding (17, 37–39). Such supervision ensures that students are involved in activities and practices that enable constructive friction between what they know and what they do not know (10, 38, 41). Consequently, students get an opportunity to translate their theoretical knowledge into practice while gradually gaining new knowledge and skills. This is crucial to the process of assimilating into the profession, hence professional socialization (13, 38). Clinical rotation starts at the first phase of the professional socialization process of medical students into the medical profession (13, 17). It is an important vehicle for students to transition from classroom-based learning to bedside learning (24, 41). Students' learning experiences during internship form their formative views about the medical profession and has a long-lasting impact on their professional development (1, 17, 38).

### Study Context and Setting

#### Medical Education and Internship Rotation in Taiwan

Taiwan has a 7-year medical program. The curriculum is divided into three parts; liberal arts, followed by medical science and clinical science, respectively, each lasting for 2 years. Students in the 7th year of school have to enroll in a mandatory clinical internship rotation. These students have not received their medical license. The internship includes block-rotation which required 48 credits comprising 40 compulsory credits and eight optional credits (see Table 1). The compulsory disciplines include internal medicine (12), surgery (12), pediatrics (6), gynecology/obstetrics (6), emergency medicine (2), and neurology (2). The goal of the internship rotation is to expose medical students to the clinical environment and to give them more opportunities for hands -on interaction with patients. Our study sample represents the last group of students in the 7-year medical curriculum. Following Taiwan's medical education reform aiming to reduce the curriculum to 6 years that took effect in 2013, in 2019 the first class of the 6-year curriculum graduated.

**Table 1:** Credits and disciplines of ED internship requirements.

**Disciplines**	**Credits**
**Compulsory**	
Internship in Medicine	12
Internship in Surgery	12
Internship in Obstetrics and Gynecology	6
Internship in Pediatrics	6
Internship in Emergency Medicine	2
Internship in Neurology	2
Optional*	
Internship in Ophthalmology	2
Internship in Otorhinolaryngology	2
Internship in Psychiatry	2
Internship in Dermatology	2
Internship in Rehabilitation Medicine	2
Internship in Anesthesiology	2
Internship in Family Medicine	2
Internship in Pathology	2
Internship in Radiation	2
Internship in Tumor Radiology	2
Internship in Nuclear Medicine	2

^*^ED internship required 48 credits which comprises 40 compulsory credits and eight optional credits.

#### ED Internship Rotation

The study was conducted in a clinical skills and simulation center of a tertiary teaching hospital in North Taiwan. The emergency department of this medical center receives ~170,000 annual visits. Concerning the EM clinical rotation, interns are required to enroll in a 2-week block rotation. The internship consists of a 1-day orientation nested within a 2-week block internship rotation in the EM. A group consisting of five attending physicians who identify as clinical educators, came together and developed clinical scenarios that were typical of EM model of care or healthcare delivery. These were compiled to create a half-day simulation-based orientation curriculum to help introduce interns to the EM work environment. Ethical approval was obtained by the participating hospital (IRB no. 201700664B0).

### Participants, Relationship Established, and Data Collection

#### Participants

The EM orientation curriculum began running in 2017 December. Our convenience sampling recruiting strategy targeted the population of medical students who enrolled in the EM internship between 16th Dec. 2017 to 18th Feb. 2019. Attending the orientation and interviews was voluntary. The study utilized bulletin board announcement in the teaching hospital to enroll medical students. Participants will contact our research assistant autonomously and will be enrolled only if the inform consent is completely signed which means s/he understood all rights and obligations before deciding whether to participate the research or not. Participants will have the same learning and assessment opportunities in EM workplace as non-participants. Medical students may dropout of the study at any time without any explanation.The exclusion criteria include medical students who are not interested in EM pre-course simulation activities or be engaged in interviews or focus group discussions for data collection.

#### Relationship Established

Most of the researchers in this study are clinical educators in emergency and surgical workplace in Chang Gung Memorial Hospital, which located closely to Chang Gung University where most clinical trainees came from. Usually there are around 150–160 medical students apply internship rotation in Chang Gung Memorial Hospital and most of them have been encountered in teaching and learning interactivities in their undergraduate years.

#### Data Collection

A total of 151 final-year medical students participated in the pre-course high-fidelity simulation and were invited to participate in the *post-hoc* focus group discussions. There is no participant has dropped out from this study. Participants had a mean age of 25.6 (±2.5) and were composed of 100 males (66.2%). After giving informed consent, interns engaged in high-fidelity simulation training and debriefing session on the first day of the EM rotation. Learners' demographic details, technical skills assessment and perceived non-technical skills when playing a leadership role were recorded. Following the simulation activity, interns participated in a post-simulation interview. This interview aimed at gathering interns' perceptions about their experience during the simulation and the anticipated influence of simulation in shaping their experiences in the EM workplace. Interns shared their perception on the educational impact of this activity. After the interview, participants were immersed in the EM work environment and spent 2 weeks in the emergency department familiarizing themselves with the basics of EM. They joined real patient care activities and interacted with ED colleagues in EM workplace. At the end of their rotation, participants were invited back for a post-rotation focus group discussion. This post rotation focus group discussion aimed at evaluating participants' experiences across the 2 weeks including the simulation experience. They were specifically requested to reflect on the effectiveness of pre-course simulation experience on workplace learning. We hold two focus group discussions for each 2-week rotation and 50 focus group discussions totally, including 29 post-simulation focus group discussions (*n* = 149) and 21 post-rotation focus group discussions (*n* = 94). The gap between the number of post simulation focus group discussions and post rotation focus group discussions took place because of the nature of EM workplace learning, work in shifts and medical students' vacation. A detailed fous group discussion schedule is provided in Appendix A. The post-simulation interviews were conducted in the simulation room in the clinical skills center where the simulations took place, while the post-rotation focus group discussion interviews were held in a meeting room in the emergency department. All interviews were conducted by YCC and the research assistant RYLN. Each interview took ~30 min (median = 28 min). The interview questions were pilot tested and revised by the research team. A sample of the post simulation and post rotation interview questions is attached to in Appendix B. All interviews were conducted in Chinese language and audio recorded and were sent to a professional translation company for transcribed verbatim. Before sending interview audios for transcription, participants' focus groups' files were re-labeled using unique identifiers representing their group number, interview time and number of participants in each discussion (e.g., FG1-PS5 = focus group No. 1, post-simulation interview, comprises five participants in the focus group discussion; FG12-PR5 = focus group No 12, post rotation interview, comprise five participants in the focus group discussion). The interviewers have written field notes during and after the focus group discussions and all transcripts were double-checked by our authors and research assistants to ensure the quality and accuracy of our qualitative data.

### Simulation Scenarios and Setting

Five simulation scenarios were designed by clinical educators using high-fidelity model. These topics include 1. ectopic pregnancy with hypovolemic shock, 2. status asthmatics with impending respiratory failure, 3. acute ST-segment elevation myocardial infarction (STEMI) with presenting acute chest pain, 4. intracranial cerebral hemorrhage with seizure attack, 5. acute pulmonary embolism with presenting dyspnea and desaturation (Appendix C). Each participant has an opportunity of being a leader in charge of the simulated patient and had the opportunity to interact with staffs in the simulated workplace. Some participants were also invited to play the role of the simulated patient or their family member by verbally interacting with the leader. Besides the medical students and researchers, the research project has also included three non-participants for preparing the educational environment for running simulation and *post-hoc* focus group discussions.

### Data Analysis

Medical interns' technical skills during the simulation were rated as met, partial met, or not met, aiming to facilitate educators' constructive feedback and learners' reflection accordingly. For the qualitative data from the FGDs and individual interviews, we employed Clarke and Braun's thematic analysis which included the following six-steps; (1) Familiarizing with the data, (2) Generating initial codes, (3) Identifying themes (4) Reviewing themes, (5) Naming the themes (6) Writing up (42). We first familiarize ourselves with the data by reading and re-reading the transcripts to proofread them and listening to the audio-recordings while jotting down notes. By doing so we gained an overview of the participants' experiences, views and perceptions of the block clinical rotation. This was followed by a round of line-by-line hand-coding. Our unit of analysis for the coding process was excerpts with one idea. All transcripts were coded separately by two researchers, YCC and CHC using mainly inductive codes. This step aimed to generate initial codes. A third researcher, NSN, reviewed the codes and conducted another round of deductive and inductive coding. All three authors discussed the differences in their coding and tried to reach a consensus on the labeling and definitions of codes. If the three coders can't reach an agreement, then a fourth researcher MJH, was consulted. The first five interviews and transcripts were used as a primer to adjust the data approaches. After iterative coding rounds using Atlas.ti we gathered the codes together in an effort to review and interpret them. At this stage, some names of the codes were revised and others were either grouped together or separated. The process involved in this third step was an iterative process that served to group codes and form categories which later formed the basis of sub-themes and more elaborate themes. We constantly referred back to the research question to guide our interpretation. The next and fourth step involved discussing and reviewing the themes together as a team of authors to ensure that they align with the study aims and also with the representative quotes. Authors SYC and MJH first reviewed the outcomes of the analysis up to this step and commented on the codes and themes. Any disagreements were settled *via* consensus. Additional excerpts describing each theme were also compiled. Once themes were supported using quotes and excerpts from the interviews, we analyzed the content under each theme in order to assign it the right name. This is the fifth step or phase of our analysis. Finally, the sixth step involved interpretations of data on two main level; within theme and across themes to see how the data within a single theme fit together while also ensuring that the themes collectively capture the response to the research question. Data saturation will also be discussed by researchers in a way that is consistent with our research questions and the theorectical background adopted. All researchers agreed on the final analytical framework. We pulled together each theme's condensed message and used key representative quotes to support the story.

### COREQ Reporting

The COREQ (COnsolidated criteria for REporting Qualitative research) guideline has been developed by Tong et al. (43) which was produced based on a comprehensive review of 22 checklists for qualitative studies. The 32-item checklist can help researchers to report important aspects of the research team, study methods, context of the study, findings, analysis and interpretations.We have used COREQ Checklist to report the quality of this qualitative research by addressing the page number in our manuscript where we consider each of the items listed in this checklist (Appendix D).

## Results

The data analysis from the post-simulation and post-EM rotation focus group discussions resulted in the construction of four major themes;

1. Challenges in finding authentic learning experiences within the new emergency medicine environment.
2. Effectiveness of the pre-course high-fidelity simulation.
3. Limitations of EM internship rotation curriculum and pre-course simulation.
4. Suggestions for EM block internship curriculum reforms.

The next section of the article provides, a detailed explanation of each these themes. The coding tree or emerged categories can be easily identified following the subtitle number. For each theme, a representative quote highlighting key aspects of a theme or sub-theme is referenced in the text and presented on the Tables 2–5.

**Table 2:** Representative quotes of theme 1: Challenges in finding authentic learning experiences within the new environment of emergency medicine.

**Subtheme 1:**	
**Challenges inherent to the ED work environment include:**	
1. Bustling work environment	“*I think it is hard to get feedback in the ED; it is too busy. I think... It's just that learning in the emergency workplace means to have a personal set of ideas, and then to seek answers; not to wait for feedback, I think*.” (FG6-PR5)
2. Lack of learner-centered curriculum	“*…Of course, I hope that I can go to the bedside and engage [in patient care], but if there are too many patients and procedures, then those [learning opportunities] will be sacrificed.... Of course, everyone would say this situation has always been like this in the past and that it has always been this way, yes, then nothing has changed from the past…” (*FG19-PS4)
3. Pressure to multitask	*“We know that in the emergency department, there is no way for you...to focus on one task from beginning to end…When a new event arises,[you] must be able to cope with the stress of that [event]. Then you must also be able to find a way to change your mindset and immediately treat the more urgent situation.” (*FG24-PR4).
**Subtheme 2:**	
**Perceptions of authentic or meaningful participation in the EM environment involves:**	
1. Limitation of tasks to only procedural skills' tasks	*“As an intern, we are exposed to simpler procedures as they are exercises to build our skills. However, when we encounter patients, often times our seniors will take over patient care...When there are a lot of patients and lots of things to do, it makes it difficult to independently provide patient care.” (*FG7-PR3*)*
2. Creative negotiation of opportunities for meaningful participation	“*Yes, we only do procedure, so I think this is the training of intern in emergency department, that is... in the actual work of the emergency department, [we] almost never ask the patient questions. Unless you do EKG and while you are doing it for the two or three minutes, [you] just quickly ask [the patient], why did you come to the emergency room.*.”(FG3-PR5)
3. Navigation of meaningful participation	*“I will think someone is calling for an intern, and someone [needs to perform an] EKG, as if we are not doing what we are supposed to be doing? Maybe you have to get used to it, because I heard other supervisors say that the emergency department is also an environment where interruptions happen all the time, yes, there is no way [to avoid it].…You need to find your own [learning] opportunities, or you have to be really lucky to be able to follow a patient completely through their care.” (*FG20-PR2*)*

**Table 3:** Representative quotes of theme 2—Effectiveness of the pre-course high-fidelity simulation.

**Subtheme 1:**	
**As a tool for enhancing students' preparedness for the EM work culture and roles simulation provided:**	
1. An opportunity for hands-on practice	*“I think simulation is very important. We don't have a license, so it is impossible to place an order for us, and the nurses do not dare to follow us. Generally, only PGYs who are licensed doctors can do it. Simulation prepares [us] for the future in advance, it is already very much like you have a license to be responsible for opening orders in the future. This is a great help during the transition period. It is only during simulation that we have the opportunity to practice placing orders, and there is no chance in the workplace, so I don't know if it is right or not. During simulation…there are far more opportunities for clinical practice than usual.” (*FG6-PR5*)*
2. Reflective practice in a safe environment	*“I think [simulation] can be helpful. …family members, or the patient's [facial] reactions. I think we are not very good at dealing with family members' questions., most of us ignore family members, or have patients nagging us, saying that we are not paying attention to them. But I'm just thinking, in fact, realistically if we encountered such a doctor, we would be distrustful too. So that is why after simulation practice, we need to practice communicating with patients and family members during their emergency visit.” (*FG5-PS5*)*
3. Understanding of the benchmarks for complete patient care	*“I think that the biggest benefit of simulation is…to increase exposure to the clinical environment…you have to do activities like this very often in order to retain the feelings of clinical practice so that you can have a better understanding of patient-care benchmarks…just like textbook standards. When you start, even if you encounter a lot of changes [in the environment], if you know the standard process, you will feel less stressed and more confident.” (*FG8-PR5*)*
4. Enhanced sensitivity to clues	“*… [simulation] also includes skills about how the entire team works based upon task assignment and management… You might want to know the data [of vital sign] and the necessity is also highlighted in the textbook. …[In the workplace] you need to ask a nurse to help you to check vital signs., but if you don't ask for help, you won't get it. This is something that you need to rely on simulation for practice to gain faster reactions.” (*FG4-PS5*)*
**Subtheme 2:**	
**As a tool for recognizing and utilizing opportunities for participation simulation experience:**	
1. Formed a basis for meaningful observation	*“I immediately identified two STEMIs during the inspection [in the clinical setting]…But when I did it, I was actually very nervous, and then I quickly ran to the senior, because I couldn't do anything, but I was alert on what to do next. I thought I could see it [the STEMI], but when it was printed out, there were stars on the paper. At least when I saw the EKG, I knew that it was very urgent, and that I had to run the next process quickly, and then I also knew why other people were so busy. That's right, it wasn't like [I was questioning] “Why did everyone suddenly move?”* (FG16-PS5).
2. Increased confidence to ask more tailored context-based questions	“*I think it's still quite different [going to the ED rotation with and without experiencing the simulation ], so it's just one more review through simulation. It's faster to get into the situation like this. I asked the senior, what I think will be the next step, is it like this? Or ask the seniors what they would do next, what they think is the next step in this situation then think about how I was thinking about it in my head. [Without simulation] I would not know the next step, I would not know how to go next. I would have to rely on the guidance of my seniors, but if I run through it myself [during simulation], I will probably be able to walk in that direction. I just need to confirm with the seniors. I have confidence*” (FG9-PR5)
3. Increased awareness of opportunities for workplace participation	*“In the future, when you...encounter critical patients, you will not be as nervous...and when you go [to provide care] to the patient, you can be of great help. For example...if I see that my senior has intubated [the patient], I will know what the next steps are and I can help my senior by elevating their neck or something. The whole process can be a lot smoother...and in the future when you rotate to the ward, you can feel less pressure, I think.”*(FG6-PR5)
4. Increased confidence to ask for opportunities to participate	*“If we speak [about simulation]from the learner's perspective, we are more prepared. It's like when you have previewed the textbook before class, and the questions you ask will be more profound. …I believe that from the instructors' perspective, they hope to meet students who have previewed [the lesson] like this. Well, something like this can help. I think that after completing the [simulation] activity, I became less flustered and more confident… Sometimes when we go [interact with the patient]...you will feel stressed and incompetent...it can feel embarrassing asking your senior for the opportunity… once you go through this activity [the simulation], it may pique some interest and also instill more confidence...” (*FG1-PS5*)*

**Table 4:** Representative quotes of theme 3—Limitations of EM internship rotation curriculum and pre-course simulation.

**Subtheme 1:**	
**The gap between high fidelity mannequins and real patients stems from differences in:**	
1. Clinical presentation and patient acuity	“*…I think when we see a real STEMI patient, we are expected to know what the EKG will look like; what the clinical presentation will look like in a real patient... It looks easy [on the mannequin] but it is not the same... their face looks the same every time... Sometimes [real patients] will come in looking very pale and have a hard time breathing, but that does not happen during simulation*.” (FG14-PR5)
2. Tactual perception & technique	*“…during CPR, although it was said that the seniors would do it. … It would be better to at least know how much pressure to apply [during chest compression]. But I think [on a real patient] the amount of pressure needed is different from the mannequin, and it feels a lot worse in my hands.”* (FG13-PS5)
3 Addressing soft skills & teaching sensitive issue	*“That is, if a [female] patient comes to us with lower abdominal pain…at the time, the senior will think about whether to do an icon [exam]…then they have to ask the patient if they had sex recently. Maybe [yes] the patient was having intense sex and the corpus luteum had ruptured, causing this pain, and then another senior PGY, a male, he has to ask the young woman these questions while her husband is next to him. It can be embarrassing for the PGY, and then he…keeps looking back at us and said ‘how do you ask this!' It's that feeling, that there will be embarrassing situations [in the clinic].” (*FG3-PR5*)*
**Subtheme 2:**	
**Gaps between simulation- based learning and bedside learning stems from:**	
1. Variations in environment hence practice, intensity & proficiency	*“I think [to be in the clinical environment] is to concentrate, because we have to ask about [patient medical history] based on what we have learned, and then apply the skill in the clinical environment,…[however], there is still a gap between simulation and the actual clinical environment, which is more chaotic....”* (FG6-PR5)
2. Variation in feedback provided	*“If it is from a systematic point of view, I think feedback from simulation will be better. The first when comparing feedback received from simulation, in the past we would watch videos or, in the simulation environment, we are surrounded by many people. So if you are doing something right or wrong, you are more aware of your own skillset…if you interact with your seniors [in the emergency room], I think that because of this job, the seniors you encounter everyday will be different, or every day your seniors can have different personalities. In the same way, I think after working with them for several shifts, we will have a deeper understanding of how the other works, so yes I think in simulation, the feedback is more detailed…”* (FG8-PR5)
3. The way affordance is structured	*“…yes for an intern, I think this exercise in the training ward may provide some more opportunities for practice, that is, hands-on practice. But if it is in the emergency room, we [interns] are just bystanders, so it is more difficult to really apply our knowledge and assess what we [can do] and what we will really encounter in the clinic compared to what we are simulating. Our role in simulation is different compared to the reality.”* (FG6-PR5)

**Table 5:** Representative quotes of theme 4—Suggestions for EM block- internship curriculum reforms.

**Gaps in EM internship-rotation can be addressed through:**	
1. Increasing the frequency and quantity of simulation activity	“*…Yes, it is possible that when you write a case [report], you will research some information that you can then use to supplement your personal database. Then I think if the number of simulations increases, the number of cases you are exposed to increases as well. That knowledge [from each case] can become part of your personal database….”* (FG4-PR2).
2. Integration of simulation-based training into pre-existing curriculum	“*Yes, because when I was in the emergency department, we were not sure whether we would be [competent] or not. [Simulation] provided a great learning opportunity…you can receive so much feedback, then if you can incorporate all the important emergency procedures into [simulation], then this should become part of the emergency training curriculum and not just part of our orientation. That is, it should become part of the emergency training curriculum.”* (FG3-PS5)
3. Matching affordance by increasing responsibility in clinical settings	“*In the emergency department, I can't do anything. However, in other departments, such as internal medicine, we [as interns] can issue doctor's orders…some senior residents will allow us to place orders, that is allow us to be responsible for every order of the patient. So when you are in charge of this situation, you will discuss with your senior …what should be ordered…Because the sense of responsibility and pressure is on you, you will be more cautious [with your actions].”* (FG20-PR2)
4. Protecting learning time	“*…for example, if there are two patients that your supervising senior is assigned to; they may begin to assess their patients or place orders. I think observing this is really meaningful towards our learning. You will not feel guilty either because at this point in time, nor will you do an EKG or other [tasks] and then you cannot be blamed by others because this time is protected for our learning. For at least an hour, it is clearly stated that it is designated for our learning and after that we are expected to pick back up on patient care.”* (FG1-PR5)

### Theme 1: Challenges in Finding Authentic Learning Experiences Within the Context of Emergency Medicine

Our analysis of interns' learning experiences in the EM revealed that there were many challenges associated with finding authentic learning experiences that were different from other contexts. We grouped them into two sub-themes with representative quotes listed in Table 2.

#### Sub-theme 1: Challenges Inherent to the EM Environment

The first subtheme represents learning challenges participants faced during their rotation that were inherent to the EM environment. These are best portrayed by the exemplified quotes listed in Table 1. Interns' discussions revealed that the busy work environment (FG6-PR5), lack of learner-centered educational design (FG19-PS4), and pressure to multitask (FG24-PR4), are all characteristics of the Emergency work environment that make it harder for interns to find meaningful learning opportunities. Hence, affect their learning experiences.

#### Sub-theme 2: Perceptions of Authentic or Meaningful Participation in the EM Environment

The second subtheme focuses on the perceptions regarding authentic or meaningful participation. Participants talked about their frustrations over being assigned only simple procedural tasks (FG7-PS5) and how they navigated through this barrier. For instance, interns from one focus group discussed how they squeezed in conversations with patients as they were performing procedural tasks to enhance their understanding of the case (FG3-PS5). Furthermore, participants expressed the importance of learning how to navigate the challenging environment in order to use every opportunity for meaningful learning (FG20-PR2).

### Theme 2: Effectiveness of the Pre-course High-Fidelity Simulation

The main aim of the interviews was to gather interns' perceptions of the influence of pre-rotation high-fidelity simulation to their bedside learning experiences. Our analysis revealed several roles that high fidelity played in enhancing interns' experiences during their rotation. We grouped them into two sub-themes with representative quotes listed in Table 3.

#### Subtheme 1: Simulation as a Tool for Enhancing Students' Preparedness for the EM Work Culture and Roles

Interns' narratives of their experience of high-fidelity simulation as a tool for enhancing their preparedness and adaptation to new role and new work culture were overwhelmingly positive. A majority of the interns had a reverse learning experience; where they found themselves learning more from the simulation activity about EM compared to when they were immersed in the actual clinical setting. This was attributed to the fact that they were not yet licensed, thus by law had limited opportunities for hands-on practice on the EM (FG6-PR5). Participants emphasized the role of simulation as a tool for self-introspection, especially for the nuanced reflection of soft skills such as interacting with patients and family members. Therefore, it provided a safe space for reflective practice (FG5-PS5). Additionally, simulation created a level of awareness for clinical standards they have to keep in mind as they transition to the real clinical setting (FG8-PR5). Simulation prepared them for EM practice by giving them clues on what they could expect when delivering patient care. Thus, it enhanced their sensitivity to ques and prepared them to react (FG4-PS5).

#### Subtheme 2: Simulation as a Tool for Spotting and Utilizing Opportunities for Participation

Simulation increased interns' chances to spot and utilize opportunities for getting involved in clinical tasks. It also prepared them to follow what was going on in the clinical environment (i.e., situational awareness), which also helped to turn observation into meaningful learning experiences (FG16-PS5). Participants expressed how their prior simulation experiences gave them the confidence to ask more tailored questions which helped them to reflect on their own clinical reasoning (FG9-PR5). It helped interns to identify areas where they could help (FG6-PR5). They were also emboldened or empowered to actively ask for opportunity to practice (FG1-PS5), making a good impression on their seniors.

### Theme 3: Limitations of EM Internship Rotation Curriculum and Pre-course Simulation

The major limitations of this internship rotation curriculum in the EM (simulation activity and clinical rotation) cited by most participants were the gaps between simulation and real clinical practice. Participants' perspectives of the rotations' limitations were divided into two subthemes with representative quotes listed in Table 4.

#### Subtheme 1: The Gaps in the Mannequin Fidelity and Real Patients

The first perspective deals with differences in terms of fidelity. That is, how closely the simulation activities mimicked real clinical cases by utilizing the mannequin. For some participants' it was hard to overlook the differences in the mannequin and real patients. For instance, participants cannot feel and observe the acuity-specific clinical presentation on the mannequin while running the case scenario of acute myocardial infarction (FG14-PR5). They also talked about how simulation was not able to capture all the nuances and contextual factors of a clinical case. For instance, the tactual perception and techniques involved when doing chest compressions during cardiopulmonary resuscitation (CPR) in simulation was different from the one experienced in the real clinical setting (FG13-PS5). Interestingly, interns also highlighted barrier to teaching soft skills such as empathy and in addressing sensitive issue in ED workplace, such as gender and privacy issues when utilizing mannequin (FG1-PR5).

#### Subtheme 2: Gaps Between Simulation- Based Learning and Bedside Learning

The second perspective deals with gaps between simulation- based learning and bedside learning. Participants talked about the differences in the organization of the two settings (clinical vs. simulation settings), which shaped their practice experience (FG6-PR5). Participants also discussed how during the simulation they received holistic *ad hoc* feedback on their performance from knowledgeable others, but not in ED workplace (FG8-PR5). Furthermore, participants' responses revealed that they believed that the way the ED rotation course was structured (e.g., low hands-on practice affordance) limited the direct application of what they learned during simulation to the clinical settings (FG6-PR5). Hence, inhibited the role of simulation in enhancing their learning experiences in EM.

### Theme 4: Suggestions for EM Block-Internship Curriculum Reforms

Overall, participants expressed the need to tailor the EM internship curriculum toward matching the two forms of learning, simulation-based and practice in real clinical setting, so that they can complement each other. To achieve this, participants offered several suggestions through the representative quotes in Table 5.

Concerning the frequency of simulation activity, there was an overwhelming interest from most participants to have more simulation opportunities and more scenarios because they considered these to play a pivotal role in enriching their database of cases they have encountered (FG4-PR2). They advocate for simulation to become part of the EM rotation curriculum so that the two learning settings can inform each other (FG3-PS5). To match the level of affordance given to interns during the simulation activities with that of the EM, participants suggested assigning more responsibility for patient care in the EM. Although they acknowledge that this can be stressful, they reckon that, it can prompt their discussions with knowledgeable seniors (FG20-PR2). Finally, students expressed their belief that providing teaching affordance in workplace can strengthen their confidence in sense making of clinical issues within the clinical context (FG1-PS5).

## Discussion

This study set out to explore the role of high-fidelity simulation in helping interns' socialization process as they transition into the clinical workplace. We first addressed interns' perceptions of the learning opportunities and learning experiences during an EM block-internship rotation. Our analysis revealed challenges embedded in the EM work culture and model of care that inhibited students' positive and meaningful learning experiences. There were tensions in students' perceptions of the current learning opportunities provided to them such as procedural tasks and their expectations of the opportunities that could help them apply their prior knowledge into practice; thus, gradually develop their clinical skills and competencies. Our results corroborate with previous findings that report that the clinical knowledge and skills acquisition that happens during internship rotation falls short of students' expectations (3, 6, 7, 9, 41). However, unlike previous studies, our results also bring new insights about the creative ways students use to find opportunities for participating. Some of these examples include interns making use of time when there are less patients to talk to patients or taking the time when they perform procedural tasks to interact with patients in a more meaningful manner.

### Simulation as a Form of Preparation and Facilitator of Meaningful Participation

Nearly all medical students had a very positive perception of the role played by pre-course high-fidelity simulation in improving their transition, participation and overall learning experiences. From students' responses, we were able to gather that engaging in high fidelity simulation orientation activity clarified the focus of clinical work, enhanced interns' awareness and facilitated their psychological preparation for the EM work culture and environment. Simulation based education was also perceived as a facilitator of psychological safety. That is, most participants felt that simulation provided a safe space for them to practice and fail. They expressed that engaging in simulation activity reduced the ‘price of making mistakes'.

Additionally, interns reported that the simulation activities allowed them to reflect. This reflection was based on students' perception of the tasks they found too easy, or too difficult, to identify gaps in their knowledge and competencies. This process is congruous to the challenging process of finding students' zone of proximal development (ZPD) that clinical educators normally engage in to assign the right type of tasks to students. Indeed, emerging research indicates that simulation is an essential pedagogical tool for clinical teachers to explore students' ZPDs (44). Our study findings suggest that beyond helping instructors explore interns ZPDs', engaging in the simulation activity also helped students to be aware of their own ZPDs. Therefore, enhanced their sense of awareness of the knowledge and skills that they brought with them to the EM rotation; which of those would be required during their rotation and what they were missing in their repertoire of competencies.

Interns' responses revealed that another key benefit of engaging in simulation activities prior to participating in the clinical setting was that it enabled them to follow what was happening during case management. This broadened their perceptions of what constituted as meaningful participation in the real clinical setting. At times students' conceptualization of meaningful participation involved real-hands on practice. However, in a busy and overcrowded environment like the emergency medicine workplace, this conceptualization of meaningful participation was extended to include close observation when students had an idea of what was going on, could anticipate the next step and were able to spot opportunities where their limited skillsets could be put into good use. Based on this, our findings suggest that simulation is an essential tool for helping students to understand what constitutes participation opportunities in the clinical environment at their level of competency. By spotting tasks that required their level of expertise, interns were able to initiate conversation on how they could get involved in the case. This is essential for meaningful participation, a concept that has been widely associated with successful workplace-based learning and is rooted in Lave and Wegner's thesis of legitimate peripheral participation. Legitimate peripheral participation refers to what a newcomer is allowed to do as part of their learning process when they join a community of practice (14). This form of participation acts as a vehicle of learning and is normally facilitated by those playing a supervision role over the newcomer.

The question of how to ensure that trainees have adequate learning opportunities at work to help them transition into the workplace is still widely debated (5, 11). The literature suggests that affording new comers with opportunities to participate in clinical practice is the cornerstone of clinical rotation (17, 45). The importance of this supervision role is evident in a couple of theories. For instance the aforementioned theory of organizational socialization, stresses the importance of organizational efforts through organizational insiders' acceptance of newcomer as a key factor for adjustment (12, 13). Lave and Wenger (14) discussed how social and power relations are also key determinants of legitimacy in practice. That is, novice trainees need people that are invested in assigning them tasks to do that will enable them to learn. This aligns with Vygotsky's theory, where opportunities for hands on learning on manageable clinical tasks are afforded through guided practice by more ‘knowledgeable others. These people create zones of proximal development (ZPD) for newcomers by assigning newcomers with manageable tasks extracted from complex tasks to facilitate optimal learning (15). Likewise, the results of this study echo the role of organizational insiders, or senior or knowledgeable members of the community of practice.

### Study Implications

The results of this study can bring important implications on the curriculum design and integration of simulation-based learning into block clinical rotation to enhance student-centered learning. Rather than putting the burden of providing interns with opportunities to learn through participation solely on supervisors, we can equip students to spot opportunities for participating. Students can be equipped to negotiate these opportunities with their supervisors, hence empowering them to take charge of their learning process during internship rotation. Based on this, we propose that interns have a role in enhancing knowledgeable others' perceptions of them. High-fidelity pre-rotation simulation orients them to the clinical environment and culture. Giving students' an overview of the community of practice, enhancing their understanding of their ZPD and tasks that are within their scope of legitimate peripheral participation, equips them with skills to identify and negotiate opportunities to participate in the workplace. This puts students in a position where they can confidently interact with other members of the community of practice. For instance, they can proactively seek information, feedback and make an effort to interact. This may gain them acceptance by organizational insiders; a key factor in their adjustment phase of socialization (12, 13). This could potentially minimize students' lack of belonging, sense of abandonment during clinical rotation and overall perceptions of lack of opportunities to participate as reported by previous literature. Indeed, literature in medical education also supports this assumption. Building on Billett (46)'s co-participation at work theory, scholars propose that workplace based learning involves dual participation or co-participation of senior members of the community of practice and the newcomers. They posits that the degree to which workplace participation result in learning outcomes depends on both workplace affordance and the learner's desire to meaningfully engage in work activities (47, 48). The literature emphasizes that, even though the workplace can afford newcomers with opportunities to engage in social practice, and cultivate an environment where learners' needs are met with support, learning still requires individual's choice to engage and participate (49, 50). This is the essence of student-centered learning that is promoted by competency-based learning.

Pre-clinical rotation, high-fidelity simulation is a promising pedagogical tool for enhancing students' participation and engagement in block-internship rotation curriculum. Unlike previous studies our study highlights the potential role of simulation in preparing the novice to also be part of their own socialization and participation process to facilitate their transition into the different clinical environment. Thus, it is useful in their block internship rotation as they move from one specialty to another. Indeed, interns' overall perception of the activity was positive and their suggestions for improving the curriculum centered on adding more simulation sessions adding more scenarios or cases for interns and designing the curriculum in a way that the simulation and the clinical environment align and inform each other. Few adjustments have to be considered. To achieve this, some participants proposed the idea of having at least a pre-and post-rotation simulation to determine how simulation helps clinical practice and how clinical practice helps simulation. Based on this, we propose that, there is a need for additional research focusing on the development of simulated case scenarios that will improve the alignment between the simulated learning context and the clinical learning environment so that students may be able to transfer their experience from one learning context to another.

### Study Strengths and Limitations

The strength of our study is that it is based on pre-and post-rotation interviews, from a large sample of participants over the course of 14 months. All authors are senior clinicians/clinical educators or medical education researchers with extensive experience in teaching and coaching medical students in EM workplace. Thus, it provides a holistic and comprehensive view on how simulation conducted as part of students' orientation before rotating in a specialty can enhance interns' learning experiences. Our findings add to the growing body of research on how simulation-based education can be used to augment block-internship rotation. An additional strength of this study is that the result highlight how simulation can be used as a resource to empower students to negotiate and make use of participation and inclusion opportunities, an issue that has not been addressed in previous literature. However, this study has several limitations that may influence the interpretation of the results. It is a single-site study without a comparison group to which we can gauge the effectiveness of simulation against. In addition to that, due to the pre-and post-rotation focus group interview design, the number of participants in each pre-post focus group pair varied as some participants were not able to participate in the post-rotation interviews because of the nature of EM workplace learning, work in shifts and medical students' vacation. Moreover, the ideal size of a focus group is 4–12. Guidance on group size is common and seldom goes beyond a minimum of 4 (51, 52). According to Langford et al. (53), 6–10 people make it an appropriate number for a focus group discussion. The sample size (mean ± SD) in our PS-FGD and PR-FGD is 5.1 ± 1.0 and 4.5 ± 1.3. In practical consideration, our focus groups comprised homogenious participants who were under internship rotation training and the median number of the medical students is 5. Besides, we did not return transcripts to participants for comments, which means we miss a step in the qualitative analysis even the correction process or validity of the data has been completed by our authors and research assistants to ensure that the participants' thoughs were accurately represented. In light of these limitations, careful considerations should be made in applying the results to other contexts. Future studies may replicate this study in a different context and also conduct this study in multiple sites. More studies are required to explore the type of simulation scenarios that can teach interns how to build healthy interactions with knowledgeable others hence improve their opportunities to participate in clinical work.

## Conclusion

The nature of block internship rotation and complex clinical environment limits students' opportunities for meaningful participation. Our study's key findings indicate that pre-rotation orientation high-fidelity simulation activity, which offered a psychologically safe space for students to explore facets of emergency medicine and gain a contextualized understanding of the emergency work culture and environment, was essential for enhancing students' ability to identify and maximize practice affordances in real clinical practice. This facilitates interns' negotiation of legitimate peripheral participation opportunities as interns move from one community of practice to another during their block internships rotation; putting students at the center of their learning through interaction and participation process. As such, we propose that, pre- rotation simulation can be implemented as a scaffold for students to gain an overview of each specialty as they rotate into each community of practice. Hence maximizes the benefits of block internship rotation in helping medical students transition into the workplace.

## Research Team and Reflexivity

Our researchers include both male and female (NSN). YCC, CHC, and SYC are senior emergency physicians. MJH is a senior chest surgeon. NSN is a PhD student. YCC, MJH, and CHC have completed Master of clinical education. CHC has also completed Master and PhD training in the field of Biostatistics and Epidemiology. NSN has completed Master degree of science and started her PhD study, SYC is studying Master degree of medical education. Interviewer YCC is a senior emergency physician, clinical educator and have more than 10 year of simulation-based teaching experience. Another interviewer RYLN has social science training background. Both interviewers have interests in issues about medical students' preparedness and transition. Our researchers have experience and training in medical education and also in medical education research. All of them are active members of Chang Gung Medical Education Research Center and regularly engaged in undergraduate and graduate education research discussion and collaboration.

## Data Availability Statement

The original contributions presented in the study are included in the article/Supplementary Material, further inquiries can be directed to the corresponding author/s.

## Ethics Statement

Ethical approval for this study was granted by the Chang Gung Medical Foundation Institutional Review Board (IRB no. 201700664B0). The participants provided their written informed consent to participate in this study.

## Author Contributions

YCC: conceptualization, methodology, formal analysis, writing—original draft, writing—review and editing, supervision, project administration, and funding acquisition. NSN: formal analysis, data curation, writing—original draft, and writing—review and editing. SYC: formal analysis and writing—review and editing. MJH: formal analysis, investigation, data curation, and writing—review and editing. CHC: conceptualization, methodology, formal analysis, resources, writing—original draft, writing—review and editing, supervision, and funding acquisition. All authors contributed to the article and approved the submitted version.

## Funding

The authors also acknowledge the support provided the funding from the Chang Gung Medical Foundation (Grant Number-CDRPG1G0041).

## Conflict of Interest

The authors declare that the research was conducted in the absence of any commercial or financial relationships that could be construed as a potential conflict of interest.

## Publisher's Note

All claims expressed in this article are solely those of the authors and do not necessarily represent those of their affiliated organizations, or those of the publisher, the editors and the reviewers. Any product that may be evaluated in this article, or claim that may be made by its manufacturer, is not guaranteed or endorsed by the publisher.
