# Supplementary material for: Exploring the Impact of Pre-course High-Fidelity Simulation on Professional Socialization of Medical Students in Emergency Medicine Internship Rotation—A Qualitative Approach
Source: Front Med (Lausanne). 2022 Jun 30;9:933212. doi: 10.3389/fmed.2022.933212 (PMC9280693; doi:10.3389/fmed.2022.933212)
Supplement: Supplementary file 1 [file Table_1.DOCX]

Appendix A- Schedules of Focus group discussion

| FG^*^ number | Number of FG  N=151 | Number of PS-FGD^#^  N=149 | Date of PS-FGD | Number of PR-FGD^&^  N=94 | Date of PR-FGD |
| --- | --- | --- | --- | --- | --- |
| FG1 | 5 | 5 | 16-Dec-17 | 5 | 27-Dec-17 |
| FG2 | 2 | 2 | 1-Jan-18 | 2 | 11-Jan-18 |
| FG3 | 5 | 5 | 16-Jan-18 | 5 | 25-Jan-18 |
| FG4 | 6 | 5 | 1-Feb-18 | 2 | 8-Feb-18 |
| FG5 | 5 | 5 | 21-Feb-18 | - | miss |
| FG6 | 5 | 5 | 1-Mar-18 | 5 | 15-Mar-18 |
| FG7 | 5 | 5 | 16-Mar-18 | 3 | 31-Mar-18 |
| FG8 | 5 | 5 | 1-Apr-18 | 5 | 17-Apr-18 |
| FG9 | 6 | 6 | 16-Apr-18 | 5 | 26-Apr-18 |
| FG10 | 7 | 7 | 1-May-18 | 7 | 15-May-18 |
| FG11 | 5 | 5 | 16-May-18 | 5 | 23-May-18 |
| FG12 | 6 | 6 | 1-Jun-18 | 6 | 14-Jun-18 |
| FG13 | 5 | 5 | 16-Jun-18 | 5 | 28-Jun-18 |
| FG14 | 5 | 5 | 1-Jul-18 | 5 | 12-Jul-18 |
| FG15 | 5 | 5 | 16-Jul-18 | 5 | 26-Jul-18 |
| FG16 | 6 | 5 | 1-Aug-18 | 5 | 14-Aug-18 |
| FG17 | 6 | 6 | 16-Aug-18 | - | miss |
| FG18 | 5 | 5 | 1-Sep-18 | - | miss |
| FG19 | 5 | 4 | 16-Sep-18 | - | miss |
| FG20 | 4 | 4 | 1-Oct-18 | 2 | 12-Oct-18 |
| FG21 | 5 | 5 | 16-Oct-18 | 5 | 1-Nov-18 |
| FG22 | 6 | 6 | 1-Nov-18 | - | miss |
| FG23 | 7 | 7 | 16-Nov-18 | - | miss |
| FG24 | 6 | 6 | 1-Dec-18 | 4 | 13-Dec-18 |
| FG25 | 4 | 4 | 16-Dec-18 | 4 | 28-Dec-18 |
| FG26 | 4 | 4 | 2-Jan-19 | 4 | 11-Jan-19 |
| FG27 | 5 | 5 | 16-Jan-19 | 5 | 28-Jan-19 |
| FG28 | 7 | 7 | 1-Feb-19 | - | miss |
| FG29 | 4 | 4 | 18-Feb-19 | - | miss |

^*^FG: Focus group; ^#^PS-FGD: post-simulation focus group discussion; ^&^PR-FGD: post rotation focus group discussion
