# Supplementary material for: Exploring the Impact of Pre-course High-Fidelity Simulation on Professional Socialization of Medical Students in Emergency Medicine Internship Rotation—A Qualitative Approach
Source: Front Med (Lausanne). 2022 Jun 30;9:933212. doi: 10.3389/fmed.2022.933212 (PMC9280693; doi:10.3389/fmed.2022.933212)
Supplement: Supplementary file 2 [file Table_2.DOCX]

Appendix B - Interview Questions

| **Themes** | **Interview Questions** |  |
| --- | --- | --- |
| 1^st^ Interview | Post-simulation interview |  |
| Opening discussion | Do you think it was useful to go through the simulation orientation training? If so, how did it? |  |
| What was your impression of emergency medicine | What was your impression of emergency medicine before attending the simulation orientation training? |  |
|  | What did you learn about emergency medicine after attending the simulation orientation training?  What is your impression of emergency medicine after attending the simulation orientation training? |  |
|  | Did the simulation help you to understand the functions and operations of the emergency department? If so, how did it? |  |
| Learning need and expectation | Did you have any learning expectations before attending the simulation orientation training? If so, what were they? |  |
|  | After attending the simulation orientation training, did it help you identify any learning needs? |  |
| Experience of role play in the simulation | You were playing different roles during the simulation orientation training (as a physician, patient and/or patient’s family member). What did you learn from taking on these different roles, if any? |  |
|  | You had to deal with / manage the response of the family member(s). What did you learn from having to manage their response, if any? |  |
|  | There were simulated family members present during the simulation orientation training. Did you find the inclusion of these family members useful for your learning during the simulation orientation training? If so, how did it? |  |
| Simulation-based learning content | Do you think the scenarios that you went through during the simulation training today were useful to help you in your training in the emergency department? |  |
| Leadership experience | You took on a leadership role during the scenarios. Did the experience benefit you? If so, how did it? |  |
| Sensitive issues | You/your fellow trainee was asked to handle sensitive issues during the simulation (i.e. taking sex history). What did you learn from this experience, if any? |  |
| Application | How do you think the simulation orientation training will prepare you for the actual clinical practice, if any? |  |
| Debriefing and feedback | What do you think of the debriefing that was done after each simulation exercise? What did you learn, if any / do you think the debriefing session helped in your learning?   - your own reflection - your fellow trainee’s reflections - teacher’s debriefing |  |
| Experience on technology-enhanced learning | Did the use of the computerised simulation mannequin technology help you learn during the simulation orientation training? If so, how did it? |  |
| Confidence | On a scale of 1 to 10, how confident did you feel about going to work in the emergency department before the simulation orientation training, 10 being the most confident? |  |
|  | On a scale of 1 to 10, how confident did you feel about going to work in the emergency department after the simulation orientation training, 10 being the most confident? Why was there a change, if any? |  |
| Anxiety | On a scale of 1 to 10, how anxious did you feel about going to work in the emergency department before the simulation orientation training, 10 being the most confident? |  |
|  | On a scale of 1 to 10, how anxious did you feel about going to work in the emergency department after the simulation orientation training, 10 being the most confident? Why was there a change, if any? |  |
| Thinking process | Did the simulation orientation training help you think about how you should think / react during the actual clinical setting? Did it help you shape your thinking process? If so, how did it? |  |
| Summary | What are some ways in which you think the simulation orientation training has benefitted your learning? |  |
|  | Is there anything that you had hoped you could have learn during the simulation orientation training which you feel you did not get to learn? |  |
| 2^nd^ Interview | Post-rotation interview | |
| Post-simulation feelings | Can we ask you a bit about the simulation orientation training, after you left the simulation orientation training, and just as you started going through the actual rotation training, did you still remember your experience during the simulation training. Did it help you in any way along the way? | |
| Differences between actual and simulated training | Comparing the simulation training with the actual training, how do you think they helped you understand the work at the emergency department? Do you think there is a difference? / What are the differences? | |
|  | Comparing the actual orientation training with the simulation training, what do you think are the differences in what you have learnt? | |
|  | Do you have an example which you share that you think can illustrate what the differences are between learning in a simulated setting vs an actual setting? | |
| Application to clinical practice | Now that you have gone through the actual clinical practice, what do you think you learnt during the simulation orientation training that helped you during your clinical practice, if any? | |
|  | Were there times that you looked back over the past two weeks during the actual clinical practice that you thought about the simulation orientation training? What did you think about? | |
| Learning time | What do you think of the learning time during your actual training? Did you think the pace of learning during the actual training was good? | |
| Debriefing | Were there times you were taken aside by yourself or in a group to be debriefed during the actual training? If yes, how would you compare it with the simulation training? If no, do you think the orientation training was helpful in this sense? | |
| Opportunity to be leader | You had the opportunity to be a leader during the simulation training but you did not get to play this role during the actual training. Do you think that made a difference? | |
| Sensitive issues | Did you come across any sensitive issues during the actual orientation, or sensitive issues that you faced during the simulation training? What was the experience like? | |
| Patient interaction | Did the simulation orientation training help you during patient interaction in the actual clinical setting? If so, how did it? | |
| Empathy | Some of you mentioned that the simulation training helped you to have more empathy. Do you think it helped you during the actual orientation training? How did it? | |
| Confidence | Some of you mentioned that the simulation training made you more confident about going to work in the emergency department. Did you think it helped you during the actual orientation training? How did it? | |
| Adequacy of simulation training | If you could try to imagine, if you had gone through the actual orientation with or without the simulation training, do you think it would have made a difference? | |
| Inadequacy of simulation training | What did you learn during the actual clinical practice which you felt you could not learn during the simulation orientation training? | |
