# Supplementary material for: Exploring the Impact of Pre-course High-Fidelity Simulation on Professional Socialization of Medical Students in Emergency Medicine Internship Rotation—A Qualitative Approach
Source: Front Med (Lausanne). 2022 Jun 30;9:933212. doi: 10.3389/fmed.2022.933212 (PMC9280693; doi:10.3389/fmed.2022.933212)
Supplement: Supplementary file 3 [file Table_3.DOCX]

Appendix C: Case scenarios of pre-course simulation activities

| A. Scenario Title: **Ectopic pregnancy with hypovolemic shock** |
| --- |
| Brief summary of case |
| A 26-year-old female presents to the emergency department for lower abdomen pain with nausea and vomiting. Her breathing was effortless. Initial assessment at triage disclosed lower blood pressure and tachycardia. The pain began 6 hours ago and reached its peak intensity 30 mins before arrival. She also complained of malaise, dizziness and diaphoresis. History is provided by herself and her father accompanied her. She denied fever, dyspnea, cough, vaginal bleeding, tarry stool, headache and blurred vision. Her last menstrual cycle ended approximately 7 weeks ago, while her cycles are typically regular. The lady will ultimately be found to have a ruptured ectopic pregnancy with hypovolemic shock. The medical student is expected to demonstrate clinical skills essential for differential diagnosis, including point-of-care ultrasound skill, appropriate tests order and accurately data interpretation. The student is expected to manage the unstable clinical condition and parameters of her vital sign will become normalized after appropriate actions were conducted such as large amount fluid resuscitation or even blood transfusion, etc. Consultation of gynecologist and hand-over are needed before the scenario ended. |
| Expected student behavioral outcomes |
| 1. Check vital sign and level of consciousness 2. Apply Oxygen/ IV set/ Monitor 3. Focused history taking (vaginal bleeding/ last menstrual period (LMP)/ sex exposure) 4. Normal saline fluid boluses 5. Pain management 6. Perform Focused assessment with sonography for trauma (FAST) and imaging reading 7. Order Laboratory tests (CBC/DC, UA, Biochemistry) 8. Order pregnancy test and accurately interpret test results 9. Consultation of the duty obstetrician-gynecologist 10. Blood transfusion 11. Demonstrate ability to provide a concise, through a handover report |
| B. Scenario Title: **Status asthmatics** with impending respiratory failure |
| Brief summary of case |
| A 36-year-old man was accompanied by his wife to the emergency department due to chest tightness, dyspnea and dry cough. Due to the patient's shortness of breath, he could only speak intermittently. The triage nurse immediately checked the vital signs and found that the ECG monitor displayed sinus tachycardia and finger pulse oximeter parameter was around 90%. History is provided by the patient with assistance by his wife knowing that he had a history of asthma in the past, often required the occasional bronchodilator spray inhalation, and had been admitted to an intensive care unit for respiratory failure. Due to his office being decorated recently, he has been coughing for a few days. Focused physical examination and preliminary treatment were expected to be given after initial assessment. Unfortunately, the patient responded poorly and gradually became confused, and the finger pulse oximeter parameter became less than 90%. The initial report of ABG showed hypercapnia and acidosis. Medical students are expected to be able to perform airway assessment, prepare for endotracheal intubation after giving medications for rapid sequence intubation (RSI), perform intubation after self-protection, interpret blood and imaging reports after intubation, update family member the critical condition, and then conduct telephone handover with colleagues in the intensive care unit for disposition. |
| Expected student behavioral outcomes |
| 1. Apply Oxygen/ IV set/ Monitor 2. Focused history taking 3. Focused physical examination (Wheezing breathing sound) 4. Order arterial blood gas (ABG) sampling and accurately analyzes ABG findings 5. Nebulizer inhalation/ MgSO4 infusion/ Steroid infusion 6. Decision of Intubation 7. Self-Protection 8. Appropriate airway evaluation and Bag-Valve-Mask use 9. Administer medications for rapid sequence intubation (RSI) 10. Appropriately use of laryngoscope 11. Post-intubation evaluation and endotracheal tube location conformation 12. Demonstrate ability to provide a concise, through a handover report with staffs in intensive care unit (ICU). |
| C. Scenario Title: **Acute ST-segment elevation myocardial infarction** (STEMI) with presenting acute chest pain |
| Brief summary of case |
| A 69-year-old woman has suffered from chest pains with cold sweats and fatigue since last night. The patient was overweight, had a long-term smoking habit and a history of hypertension for more than 20 years. The patient stated that he had no recent trauma history, no fever or cough, and no abdominal pain or bloody stools. Triage station nurses has quickly evaluated the patient’s vital sign found that hypotension and bradycardia, and his electrocardiogram (ECG) disclosed inferior wall myocardial infarction. The medical student is expected to order a right-side ECG exam to assess for further evidence of right ventricular involvement. The patient is still hypotensive after oxygen, aspirin, and 500 cc normal saline fluid infusion used. Unfortunately, the patient suddenly loses consciousness and ECG monitor disclosed rhythm as ventricular fibrillation. The student is expected to perform high-quality CPR and urgent defibrillation for the patient. Return of spontaneous circulation (ROSC) was noted soon and he was sent to the cardiac catheterization lab. |
| Expected student behavioral outcomes |
| 1. Check Vital sign (shock) 2. Order ECG and accurately ECG interpretation (Inferior wall Infarction) 3. Order Right side EKG and accurately Interpretation 4. Consultation of CV expert for percutaneous coronary intervention (PCI) 5. Appropriate fluid resuscitation for RV filling pressure and cardiac output. 6. Communicate with the patient and updates the family 7. Immediate defibrillation for cardiac arrythmia event (ventricular fibrillation) 8. Timely perform cardiopulmonary cerebral resuscitation (CPCR) 9. Identify return of spontaneous circulation (ROSC) and check vital sign 10. Demonstrate ability to provide a concise, through an accurate handover report with staffs in ca cardiac catheterization room. |
| D. Scenario Title: Pulseless electrical activity of a transferred patient with **dislodge of endotracheal tube** from other hospital |
| Brief summary of case |
| A 69-year-old male suffered from sudden headache, vomiting and progressive drowsiness. Brain computed tomography examination at the regional hospital disclosed left cerebral cerebral hemorrhage. The emergency physician performed intubation to protect airway and he was immediately transferred to the emergency department you serve in a medical center. According to the EMT, the level of consciousness become worse on the way. Medical students are expected to take focused medical history from EMT and his family accompanied, reassess the patient's vital signs, and confirm that all tubes, lines and attached machines are functioning properly. The patient's endotracheal tube dislodged during the transfer process, and the ECG showed pulseless electrical activity (PEA). The medical student was expected to perform essential skills for patient transfer handover, high-quality CPCR and re-intubation to establish the airway. Brain CT was scheduled to assess the progression of the intracerebral hemorrhage, and a neurosurgeon was consulted. |
| Expected student behavioral outcomes |
| 1. Demonstrate ability to conduct focused history taking for the transferred patient 2. Ensure that all tubes, attachments, monitors, attached machines and lines are placed properly 3. Demonstrate ability to check vital sign and perform focused physical examination (pulseless) 4. Identify pulseless electrical activity (PEA) 5. Activate and perform high quality cardiopulmonary cerebral resuscitation (CPCR) 6. Identify dislodge of the endotracheal tube and accurately perform re-intubation 7. Consult respiratory therapist for ventilator use and setting 8. Order a brain CT Image and accurately reading (intracranial cerebral hemorrhage) 9. Team dynamic skills 10. Administer Medications accurately (e.g., epinephrine) 11. Identify return of spontaneous circulation (ROSC) 12. Reassess Vital sign 13. Consult neurosurgeon |
| E. Scenario Title: **Acute pulmonary embolism** with presenting dyspnea and desaturation |
| Brief summary of case |
| A 72-year-old man was sent to the emergency room with sudden dyspnea and chest tightness. According to family witnesses, the patient felt briefly fainted when he was awake and became cyanotic, and then continued to have difficulty breathing. Vital sign and quick look evaluation at the triage station disclosed tachycardia, desaturation, borderline blood pressure of 95/60 mmHg, and swelling of the patient's right lower extremity. In the focused history taking, the patient had no history of tumors in the past, but had recently undergone spinal surgery. After the operation, he had been in bed for nearly two weeks due to pain intolerance and was inconvenient to move. Students are expected to ask about dyspnea related medical history, perform point-of-care ultrasound, and prescribe CT scans for pulmonary embolism after correct interpretation of test results. After CT scan proved pulmonary embolism, the simulation will end after a consultation was conducted. |
| Expected student behavioral outcomes |
| 1. Check Vital sign and patient identification 2. Focused physical examination 3. Focused history taking (deep vein thrombosis, surgery, bedridden, hemoptysis, active cancer, etc.) 4. Oxygen/ IV set/ Monitor 5. Assessment of Wells score 6. Order EKG and accurately interpret data 7. Order CXR, CBC-DC, Chemistry and ABG test and demonstrate ability to accurately interpret data 8. Point-of-care ultrasound for collecting evidence for deep vein thrombosis or pulmonary embolism. 9. Order CT angiography (CTA) for pulmonary embolism 10. Communication with the patient and update his family 11. Demonstrate ability to accurately interpret CTA image 12. Evaluation the indication for Alteplase (rt-PA) 13. Consultation of CV doctor or cardiovascular surgeon |
